# A Move in the Right Direction

**Published:** 1873-09

**Authors:** 


### *A Move in the Right Direction.*

At the recent meeting of the State Medical Society the following resolution was introduced by Dr. Ira Oatman, of Sacramento:

"*Resolved*, That it is the duty of, and we hereby recommend to, the Legislature of California to pass a law making it a misdemeanor for any person, for any purpose whatever, who is not a graduate of some institution of learning authorized by law to confer the degree of 'Doctor of Medicine,' who shall place before or after his or her name, in any manuscript, label, wrapper, card, handbill, circular, newspaper, pamphlet, magazine, book, or any advertisement, the word 'Doctor' or the abbreviation M.D. or Dr., or any others signifying directly or constructively that the person is a graduate of such an institution, or who shall authorize or sanction the same by others in his or her interests; and that any person found guilty of such misdemeanor shall be punished by a fine of not less than ——— dollars, or imprisonment for not less than ——— years, or by both such fine and imprisonment."

—*Western Lancet.*

